# Lipocalin‐2 Restores Soluble Guanylyl Cyclase‐Dependent Dilation of the Afferent Arteriole After Renal Transplantation or Ex Vivo Hypoxia/Reoxygenation in Mice

**DOI:** 10.1111/apha.70077

**Published:** 2025-07-04

**Authors:** Liang Zhao, Minze Xu, Anna Maria Pfefferkorn, Cem Erdogan, Hubert Schwelberger, Pinchao Wang, Pratik Hemant Khedkar, Marc Eigen, Falk‐Bach Lichtenberger, Rusan Catar, En Yin Lai, Felix Aigner, Pontus B. Persson, Igor Maximilian Sauer, Andreas Patzak, Muhammad Imtiaz Ashraf

**Affiliations:** ^1^ Institute of Translational Physiology, Charité – Universitätsmedizin Berlin Corporate Member of Freie Universität Berlin and Humboldt‐Universität zu Berlin Berlin Germany; ^2^ Department of Surgery, Experimental Surgery, Campus Charité Mitte | Campus Virchow Klinikum, Charité – Universitätsmedizin Berlin, Corporate Member of Freie Universität Berlin Humboldt‐Universität zu Berlin Berlin Germany; ^3^ Department of Visceral, Transplant and Thoracic Surgery Medical University Innsbruck Austria; ^4^ Department of Nephrology and Internal Intensive Care Medicine Charité – Universitätsmedizin Berlin, Corporate Member of Freie Universität Berlin, Humboldt‐Universität zu Berlin Berlin Germany; ^5^ Department of Physiology, School of Basic Medical Sciences Zhejiang University School of Medicine Hangzhou China; ^6^ Department of Surgery Krankenhaus der Barmherzigen Brüder Graz Austria

**Keywords:** hypoxia/reoxygenation, Lipocalin‐2, microvascular function, mouse kidney transplantation

## Abstract

**Aim:**

Dilatory microvascular function is impaired in ischemia/reperfusion injury in the kidney. Nitric oxide independent activators of soluble guanylyl cyclase (sGC) provide renal protection by dilating microvessels and preserving perfusion, but their efficacy declines after severe hypoxia. This study explores whether lipocalin‐2 (Lcn2), a key iron‐transporting protein, can restore the sGC‐mediated dilation in mouse afferent arterioles (AAs) after hypoxia/reoxygenation (*H*/*R*) and kidney transplantation.

**Methods:**

Dilation of isolated, angiotensin II (Ang II) pre‐constricted, AAs was induced by application of sGC activator cinaciguat after pre‐constriction with Ang II following *H*/*R* (*H*: 30 min, *R*: 10 min ± holo‐rLcn2, apo‐rLcn2, deferoxamine) and syngeneic kidney transplantation (cold ischemia: 30 min or 5.5 h, reperfusion: 20 h ± holo‐rLcn2) in C57BL/6 mice. To corroborate the dilatory function at the organ level, vascular relaxation was assessed using an isolated mouse kidney perfusion system.

**Results:**

Dilation of AAs was impaired following *H*/*R*. Pretreatment with holo‐rLcn2 (iron‐bound) preserved dilation, whereas apo‐rLcn2 (iron‐free) had no effect. The reversal of holo‐rLcn2's effect by deferoxamine confirmed the role of iron. AAs from kidney transplants showed reduced dilation compared to sham‐operated controls, with greater impairment following prolonged ischemia. Treatment with holo‐rLcn2 significantly improved dilatory function after extended cold ischemia (5.5 h), restoring it to levels seen with shorter ischemia (30 min). Ex vivo perfusion of the isolated mouse kidney with holo‐rLcn2 enhanced cinaciguat‐induced vascular relaxation, confirming its beneficial effect at the organ level.

**Conclusion:**

This study identifies a novel role for holo‐rLcn2 in preserving renal vascular function post‐*H*/*R* and kidney transplantation, apparently by upholding iron levels in vascular cells.

## 1 Introduction

The incidence of end‐stage renal disease (ESRD) is rising globally, seriously affecting patients' quality of life and overall health. At present, kidney transplantation is the most effective treatment for ESRD. Renal ischemia/reperfusion injury (IRI), resulting from interruption of blood supply and its following re‐establishment, is an important factor in the damage and functional recovery of the transplanted kidney [1]. Donor organ shortage has necessitated the transplantation of older and high‐risk deceased organs, which are even more vulnerable to IRI and delayed graft function [2, 3]. Therefore, ways to prevent or enhance recovery from IRI are urgently needed.

Lipocalin‐2 (Lcn2), also known as neutrophil gelatinase‐associated lipocalin (NGAL), is a glycoprotein involved in various physiological and pathological processes. It is a sensitive biomarker in the early stage of renal damage following acute kidney injury and renal transplantation [4, 5, 6, 7]. Lcn2 functions primarily in transporting small hydrophobic molecules, particularly iron, through its binding to siderophores. Depending on whether or not it is bound to iron, it may deliver or sequester iron in the targeted cells and exert corresponding biological effects. The iron‐loaded form of recombinant Lcn2 (holo‐rLcn2) delivers iron to the targeted cells and protects them from apoptosis, while the iron‐deficient form (apo‐Lcn2) depletes cellular iron and induces apoptosis [8]. Similarly, Lcn2 has been shown to ameliorate IRI by reducing apoptosis of tubular epithelial cells and by stimulating their proliferation, which seems to depend on the delivery of iron [9, 10, 11]. Using a mouse model of kidney transplantation, we too demonstrated a protective function of holo‐rLcn2 during the early phase of rejection [12]. Impaired renal microcirculation is an important determinant of IRI in the kidney transplant [13]. However, whether Lcn2 can influence renal microvascular function and preserve renal perfusion following IRI has not been shown so far.

Renal microvascular tone increases after ischemia/reperfusion (I/R) due to an impaired balance of vasoconstrictor and ‐dilator activity [14, 15, 16, 17, 18]. Renal microvascular tone depends considerably on the dilatory action of nitric oxide (NO) [19, 20, 21]. NO produced by endothelial (eNOS) and neuronal (nNOS) NO synthase diffuses into vascular smooth muscle cells and activates soluble guanylyl cyclase (sGC), thereby increasing cGMP. Latter relaxes vascular smooth muscle cells by protein kinase G (PKG)‐mediated mechanisms [11, 22]. I/R‐induced oxidative stress impairs NO‐sGC signaling and consequently disrupts vascular function [23, 24]. Under situations of reduced NO availability and sGC oxidation, vascular dilation can be achieved by synthetic sGC activators like cinaciguat and Bay 60‐2770 [25]. They have been shown to improve renal function in several kidney disease models [26, 27, 28, 29]. The protective effect of Bay 60‐2770 includes renal microvascular function and blood flow control, strongly pointing to the preservation of the sGC‐mediated dilatory signaling [29]. However, in vitro experiments showed insufficient dilation of afferent arterioles (AAs) by cinaciguat after severe hypoxia/reoxygenation (*H*/*R*), suggesting higher sensitivity and impaired sGC‐cGMP‐PKG‐mediated vasodilatory function of AAs under these conditions [15].

In this study, we aimed to investigate whether rLcn2 can preserve cinaciguat‐induced dilation of renal AAs under experimental *H*/*R* conditions and after mouse kidney transplantation. Moreover, we functionally evaluated the effect of rLcn2 and cinaciguat on renal perfusion by determining vascular relaxation in the intact mouse kidney using an isolated machine perfusion system. We hypothesize that holo‐rLcn2 promotes the sGC‐cGMP‐PKG‐mediated relaxation of AAs by protecting renal microvascular smooth muscle cells from IRI damage.

## 2 Methods

### 2.1 Animals

Male C57BL/6 mice (*n* = 181) were used for all experiments. Further details, including their weights, the number of mice used per experiment, and exclusion from each analysis, are presented in a CONSORT‐like diagram (Figure S1). The animals were purchased from Janvier (Le Genest St Isle, France) or Charles River Laboratories (Sulzfeld, Germany) and housed under standard conditions, with ad libitum access to food and water. All animal procedures were performed following the directive 2010/63/EU, the German Tierschutz‐Versuchstierverordnung, and were approved by the Regional Ethics Committee for Animal Research (Landesamt für Gesundheit und Soziales Berlin, approval number: G0236/18).

### 2.2 Isolation of Mouse Afferent Arterioles

To isolate AAs, C57BL/6 mice were euthanized by cervical dislocation under deep isoflurane anesthesia. Left kidneys were harvested and carefully sliced along the corticomedullary axis. Renal AAs were isolated along with glomeruli by using sharp forceps, then pretreated and exposed to *H*/*R* or normoxia according to specific protocols before transferring to a perfusion chamber, mounted on the stage of an inverted microscope, containing Dulbecco's modified Eagle's medium (DMEM; Gibco, Paisley, UK) supplemented with 0.1% bovine serum albumin (BSA; Carl Roth GmbH, Karlsruhe, Germany).

### 2.3 Perfusion of Isolated Afferent Arterioles

AAs were mounted in the perfusion chamber with the help of two handmade holding pipettes. One holding pipette fixed the free end of the AA and the other one fixed the glomerulus. A perfusion pipette was inserted into the AA holding pipette and advanced into its lumen. The AA was then perfused with DMEM, supplemented with 1% BSA, in an orthograde manner at 100 mmHg pressure and 50 nL/min flow rate. The vessels were allowed to acclimatize for 10 min after establishing the perfusion. The temperature of the perfusate and bath solution was maintained at 37°C during the whole experiment. All experiments were performed within 2 h after the mouse had been euthanized. The viability of the vessels was tested by the short‐term application of KCl (100 mM) at the beginning and end of the experiment. Only the vessels that showed full and sustained constriction with KCl treatment were used for the analysis.

### 2.4 Measurement of Afferent Arteriolar Diameter

Perfused AAs were continuously monitored on the computer screen using a video camera and software (Moticam 2.0, Motic Asia, Hong Kong). Luminal diameter served for the estimation of arteriolar tone, and reactivity was measured using the free software Image J, as previously described [14]. Luminal diameter of each AA was measured in five consecutive images taken every second, and the calculated mean thereof was used to determine dose–response relationships. The measurements were conducted in a blinded manner to minimize bias. The experimenters performing the assessments were unaware of the experimental group to which the luminal diameter of the AAs being measured belonged. Measurements were performed at intact and most reacting parts of an arteriole.

### 2.5 Protocols

Series A: The isolated AAs were first subjected to normoxia (40 min) or *H*/*R* (*H*: 30 min + *R*: 10 min). For the induction of hypoxia, AAs were incubated in a hypoxia chamber (Whitley H35 “Hypoxystation” Meintrup DWS Laborgeräte GmbH, Herzlake, Germany) under 0.1% O_2_, 5% CO_2_, 94.9% N_2_ at 37°C for 30 min. For reoxygenation, the AAs were subsequently incubated at 37°C for 10 min under normoxic conditions. Normoxic controls followed the same procedures, except for exposure to hypoxia. Thereafter, the vessels were mounted in the perfusion chamber and perfused with DMEM, supplemented with 1% BSA as explained above. Next, angiotensin II (Ang II; Sigma‐Aldrich, Darmstadt, Germany) was applied in increasing concentrations (10^−12^ to 10^−6^ mol/L, each for 2 min) to the bath solution and arteriolar diameter was recorded for each arteriole every 2 min. Finally, cinaciguat (BAY 58‐2667, 10^−7^ mol/L) (Bayer AG, Wuppertal, Germany) was added to the bath solution and the arteriolar diameter was recorded every 10 s over a period of 10 min.

For the analysis of the Ang II concentration–response relation, arteriolar diameters were normalized to the initial diameter after pre‐treatment. The time response of absolute diameters following maximum Ang II exposure shows a stable constriction of AAs over the period 10 min (Figure S2).

In the treatment groups, the AAs were pretreated with vehicle (PBS; 1 μL/mL), holo‐rLcn2 (1 μg/mL), apo‐rLcn2 (1 μg/mL), or deferoxamine (DFO; Sigma‐Aldrich, Darmstadt, Germany) (100 μM) before exposure to *H*/*R* or normoxia protocols. Additionally, PBS, holo‐rLcn2, apo‐rLcn2, and DFO remained present in the bath solution throughout the entire procedure.

Series B: The isolated AAs were pretreated with vehicles (PBS; 1 μL/mL, DMSO; 1 μL/mL), holo‐rLcn2 (1 μg/mL), or sGC oxidant 1 H‐[1,2,4]oxadiazolo[4,3‐a]quino xaline‐1‐ one (ODQ; 10^−5^ mol/L) (Sigma‐Aldrich, Darmstadt, Germany) [30]. After 40 min of normoxic culture, vascular constriction and dilatation analysis of AAs were performed as described in series A, with the exception that instead of cinaciguat, the NO‐releasing compound S‐nitroso‐N‐acetylpenicillamine (SNAP; 10^−3^ mol/L) (Sigma‐Aldrich, Darmstadt, Germany) was used for vasodilation. PBS, DMSO, holo‐rLcn2, and ODQ remained present in the bath solution throughout the entire procedure.

Series C: AAs were isolated from the kidneys of sham‐operated C57BL/6 mice and kidney transplants of recipient mice treated with PBS (250 μL) or holo‐rLcn2 (250 μg). The microvessels were then mounted, perfused, and subjected to vascular constriction and dilation analysis as described in Series A. Additionally, PBS (1 μL/mL) and holo‐rLcn2 (1 μg/mL) were added to the bath solution and remained present throughout the entire procedure.

### 2.6 Mouse Kidney Transplantation

Syngeneic kidney transplantations in C57BL/6 mice were performed under isoflurane inhalation anesthesia as previously described [12]. Briefly, following a midline abdominal incision, the left kidney, aorta, and inferior vena cava of the donor mouse were exposed and carefully mobilized. The kidney was flushed in situ with histidine‐tryptophane‐ketoglutarate (HTK) solution (Custodiol, Dr. Franz Köhler Chemie GmbH, Bensheim, Germany) and procured en bloc including the renal vein, renal artery along with a small aortic cuff and ureter. The kidney was stored in ice‐cold HTK solution for 30 min or 5.5 h and thereafter implanted in the left nephrectomized recipient mouse, below the level of native renal vessels. End‐to‐side anastomoses between the donor renal vessels and the recipient's abdominal aorta and inferior vena cava were performed following a knotless technique [31]. For urinary tract reconstruction, the ureter was directly anastomosed into the bladder. The recipient mice received an injection of carprofen (5 mg/kg BW) preoperatively and buprenorphine (0.05 mg/kg BW) approximately 30 min before recovery from anesthesia. After 20 h of transplantation, the kidneys were dissected and analyzed for microvascular function. The sham‐operated mice underwent a surgical procedure similar to the recipients except for renal transplantation. In the rLcn2 treatment group, 250 μg of recombinant Lcn2:Siderophore:Fe (rLcn2:Sid:Fe^3+^) was applied directly to the donor kidney during perfusion and to the recipients perioperatively (15 min before restoration of blood supply to the graft) as previously described [12].

### 2.7 Extracorporeal Machine Perfusion of Isolated Mouse Kidneys

Extracorporeal machine perfusion of the isolated mouse kidney was performed as previously described [32]. Briefly, C57BL/6 mice were first euthanized by cervical dislocation under deep isoflurane anesthesia. Immediately afterwards, the abdomen and ribcage were cut open to reveal the heart and the kidneys. To keep the blood from clogging the renal vessels, the mice were transcardially perfused with an ice‐cold physiological salt solution (PSS) (119 mmol/L NaCl, 4.7 mmol/L KCl, 1.2 mmol/L KH_2_PO_4_, 1.2 mmol/L MgSO_4_·7H_2_O, 5.5 mmol/L glucose, 25 mmol/L NaHCO_3_, and 0.625 mmol/L CaCl_2_·2H_2_O; pH 7.4). Thereafter, the kidneys were removed and placed in a petri dish containing ice‐cold oxygenated PSS. After arterial cannulation, the kidneys were transferred to a heated organ chamber and perfused with oxygenated PSS at 37°C with a peristaltic pump (Instech Laboratories Inc., PA, USA). In the initial stages of ex vivo kidney perfusion, a low flow rate (0.3 mL/min) was maintained. This allowed for the flushing of the residual blood from the kidney and prevented high perfusion pressure, which could damage the vasculature. The pressure fluctuates during this phase and takes 15–20 min to stabilize. Therefore, an equilibration phase of 20 min was implemented, after which the kidney was perfused at a constant flow rate between 0.3 and 1.9 mL/min, such that the pressure was maintained at 80–90 mmHg. The resistance of the kidney was measured with a pressure transducer (PM4; Living Systems Instrumentation) and acquired with PowerLab (AD Instruments). To induce constriction of renal microvessels, the kidney was perfused with PSS containing Ang II (10^−8^ mol/L). After reaching a plateau of renal perfusion pressure, the kidney was perfused with cinaciguat (10^−7^ mol/L) or a combination of cinaciguat (10^−7^ mol/L) and holo‐rLcn2 (1 μg/mL) to induce vascular relaxation. Relative vascular relaxation was calculated by the ratio of the pressure decrease after vessel dilation and the pressure increase after pre‐constriction. The integrity of the kidney vessels was confirmed by perfusing the kidney with K‐PSS (PSS + 123.7 mmol/L KCl) after washing out Ang II, cinaciguat, and rLcn2 with PSS.

### 2.8 Measurement of cGMP Concentration in the Perfusate

Perfusate samples were collected immediately after maximum relaxation was observed. cGMP levels in the perfusate samples were measured using a direct cGMP ELISA kit (Enzo Life Sciences, Catalog No. ADI‐901‐014, Lausen, Switzerland) according to the manufacturer's instructions.

### 2.9 RNA Isolation From Isolated Mouse Afferent Arterioles and Kidney Sections

Both kidneys from six C57BL/6 mice were used to isolate AAs. The arterioles were freed of any other biological material and snap‐frozen. The number of dissected AAs varied between 8 and 20 per animal, resulting in a pool of 118 arterioles, used altogether for RNA isolation. A total of 1.37 μg RNA was isolated from the arterioles using RNA‐Bee reagent (AMS Bio, Abingdon, UK) and Lysis Matrix D tubes (MP Biomedicals, CA, USA) following the manufacturer's instructions.

### 2.10 cDNA Synthesis and Quantitative Real Time PCR

For cDNA synthesis, 1 μg of total RNA was reverse transcribed in a 20 μL reaction volume using oligo(dT) primer and the RevertAidTM H Minus M‐MuLV Reverse Transcriptase (Fermentas GmbH, St. Leon‐Rot, Germany). Real‐time reverse transcription polymerase chain reaction (RT‐PCR) for gene expression analysis was performed with the StepOnePlus system (Applied Biosystems, CA, USA). Primers for Lcn2 receptors, low density lipoprotein‐related protein 2 (Lrp2), also known as megalin (Mm01328171_m1) and 24p3 receptor (24p3R) (Mm00480680_m1), were directly purchased as Taqman gene expression assays (Life Technologies, Carlsbad, CA, USA). Specific gene expression was normalized to the housekeeping gene Peptidylprolyl Isomerase A (PPIA) using the formula 2^−ΔCt^.

### 2.11 Western Blotting

338 AAs, isolated from 24 mice, were pooled together before lysis in 500 μL ice‐cold RIPA lysis buffer (Thermo Fisher Scientific, MA, USA), containing protease inhibitor cocktail (cOmplete; Roche Diagnostics GmbH, Germany) and sodium orthovanadate (0.2 mM). Protein content was determined with Bio‐Rad Protein Assay. Due to the very low total protein content (0.25 μg/mL), the sample was concentrated to almost 50 μL using an Amicon Ultra centrifugal filter (Merck Millipore Ltd., Darmstadt, Germany) to enable the loading of the entire sample (approximately 1 μg/50 μL) for analysis. Proteins were separated by 7.5% SDS‐PAGE and transferred to nitrocellulose membrane (GE Healthcare, Uppsala, Sweden). The membranes were blocked for 1 h at room temperature with 5% skim milk (Applichem, Darmstadt, Germany) and 1% bovine serum albumin (SERVA, Heidelberg, Germany), dissolved in TBST (50 mM TRIZMA base, 150 mM NaCl, pH 7.5 adjusted with HCl, 0.1% Tween‐20). The membranes were then incubated overnight at 4°C with the following primary antibodies: anti‐Lrp2 (sc‐515772, Santa Cruz Biotechnology, CA, USA), anti‐Akt (#9272, Cell Signaling Technologies). Due to the large size of Lrp2 (~600 kDa), SDS‐PAGE was run for an extended duration, causing commonly used loading controls (e.g., GAPDH, β‐actin) to run out. Therefore, Akt (~60 kDa), which has a relatively higher molecular weight, was used as the loading control. Following incubation of the membranes for 1 h in HRP‐conjugated secondary antibodies (diluted in 5% skim milk), immunocomplexes were visualized by Supersignal West Pico Chemiluminescent substrate (Thermo Scientific, CA, USA). The original Western blot images are presented in Figure S3.

### 2.12 Preparation of Recombinant Lcn2

Mouse rLcn2 was expressed and purified to greater than 99% purity as previously described [12]. Briefly, mouse Lcn2 was expressed as a glutathione S‐transferase fusion protein in protease‐deficient strain *Escherichia coli* BL21, according to the manufacturer's instructions (GE Healthcare, Vienna, Austria). The fusion protein and Lcn2 were purified by chromatography on Glutathione Sepharose (GSTrap FF, GE Healthcare, Vienna, Austria), and by chromatography on CIM‐SO3 (BIA Separations, Ljubljana, Slovenia), respectively. The rLcn2 protein was incubated for 4 h at 4°C with an equimolar amount of enterobactin (apo‐rLcn2) or ferric enterobactin (EMC microcollections, Tübingen, Germany) (holo‐rLcn2) and dissolved in phosphate‐buffered saline (PBS) at 1 mg/mL concentration.

### 2.13 Statistical Analyses

Statistical analyses were performed using GraphPad Prism version 10.1.2 (GraphPad Software, San Diego, CA, USA). The D'Agostino & Pearson omnibus normality test was used to assess normal data distribution. Statistical significance between the two groups was calculated using Welch's *t*‐test. Repeated measurement analysis for diameter changes (time‐ and concentration‐responses) was performed using the Brunner test for non‐normally distributed data, which is a nonparametric counterpart of the two‐way ANOVA, provided by the “R” project (R: A Language and Environment for Statistical Computing; R Foundation for Statistical Computing: Vienna, Austria. Available online: http://www.r‐project.org/ [accessed on April 15, 2022]). Additionally, significance levels among different groups were validated using the Friedman test, which is a nonparametric equivalent of repeated measures ANOVA that supports Dunn's post hoc multiple comparisons. The results were quite consistent with those obtained from the Brunner test. Data were expressed as means ± standard deviation (SD) or standard error of the mean (SEM). A *p*‐value < 0.05 was considered statistically significant. Sample size estimation was based on data from our recent study [29], as no prior data were available on the effect of rLcn2 on microvessels. A significance level of 0.05 and a power of 0.95 were set, and a *t*‐test was applied. The calculation was performed using G*Power 3.1.9.7 (Universität Düsseldorf, Germany), which estimated a sample size of five per group.

## 3 Results

### 3.1 Hypoxia/Reoxygenation Impairs Cinaciguat‐Induced Dilation of Renal Afferent Arterioles

**FIGURE 1:**
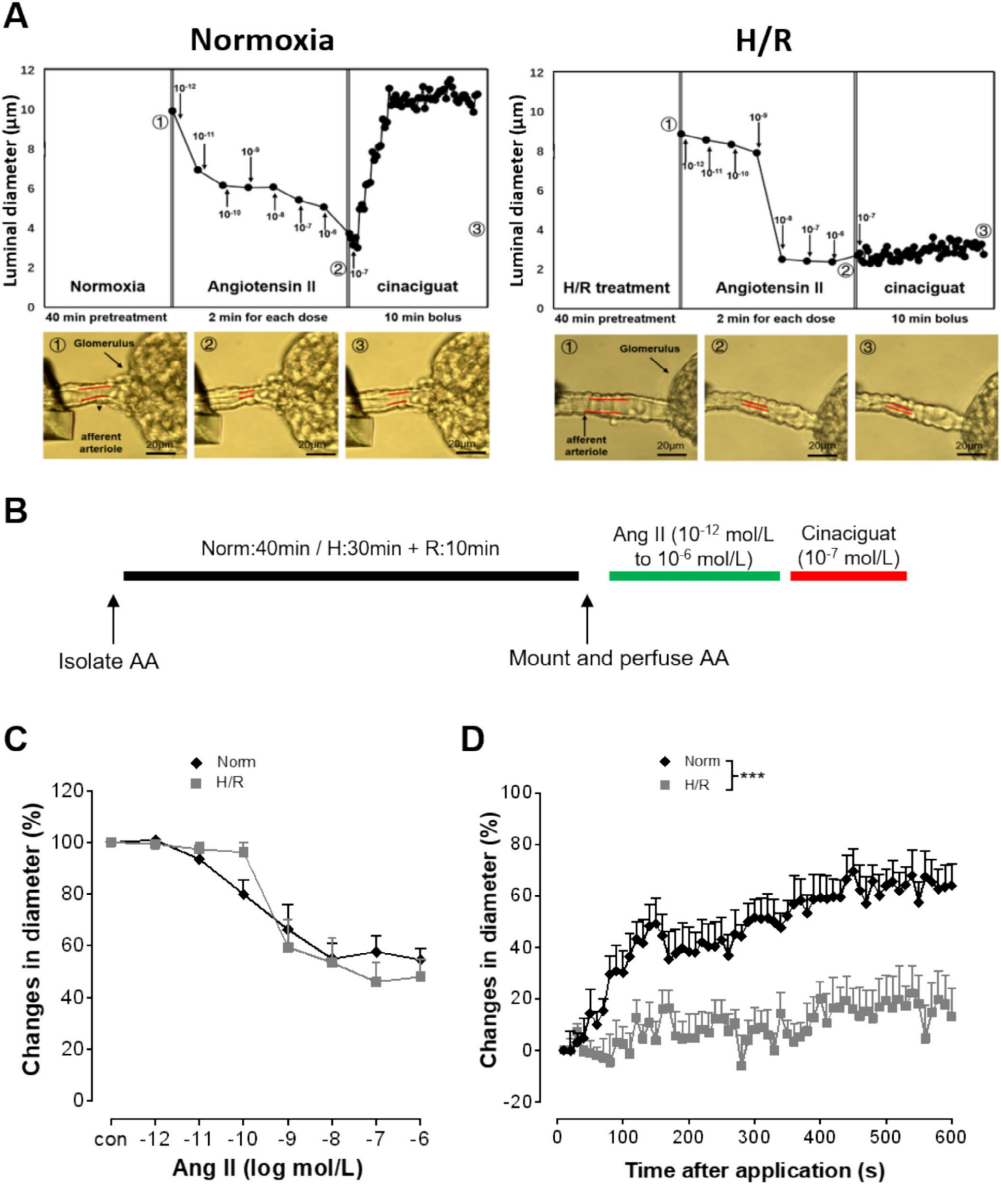
Hypoxia/reoxygenation impairs cinaciguat‐mediated dilation of mouse renal afferent arterioles. Afferent arterioles (AAs) were isolated from C57BL/6 mice and exposed to hypoxia/reoxygenation (H: 30 min/R: 10 min) or normoxia (Norm: 40 min). Angiotensin II (Ang II) was applied in increasing concentrations (10^−12^ to 10^−6^ mol/L, each for 2 min) to induce vasoconstriction and vascular diameters were recorded. Thereafter, dilation was induced by a bolus application of cinaciguat (10^−7^ mol/L) to the pre‐constricted AAs and their diameters were recorded every 10 s over a period of 10 min (A, B). The upper panels display original traces depicting changes in vascular luminal diameter during normoxia and *H*/*R*. The lower panels show representative vivid images illustrating corresponding vascular luminal diameters at different experimental stages (A). Percentage changes in the microvascular diameters during Ang II‐mediated constriction (C) and cinaciguat‐mediated dilation (D) are shown. (Mean ± SEM values; *n* = 6 Norm, *n* = 7 *H*/*R*.) ***p < 0.001 (Brunner test).

To assess the impact of *H*/*R* on cinaciguat‐induced dilation of renal AAs, the AAs isolated from C57BL/6 mice kidneys were exposed to *H*/*R* (*H*: 30 min + *R*: 10 min) or normoxia (40 min), followed by constriction and dilation with Ang II and cinaciguat, respectively (Figure 1A,B). Under normoxia, increasing concentrations of Ang II (10^−12^ to 10^−6^ mol/L, each for 2 min) resulted in constriction of isolated mouse renal AAs (Figure 1A,C). A bolus application of cinaciguat (BAY 58‐2667; 10^−7^ mol/L), soon after the Ang II treatment, resulted in dilation of the AAs (Figure 1A,D). While no significant effect of *H*/*R* was observed on the constriction response of AAs to Ang II (Figure 1C), the cinaciguat‐mediated dilation of the vessels was significantly impaired (Figure 1D).

### 3.2 Holo‐rLcn2 Ameliorates Hypoxia/Reoxygenation‐Induced Loss in the Dilation of Mouse Renal Afferent Arterioles

**FIGURE 2:**
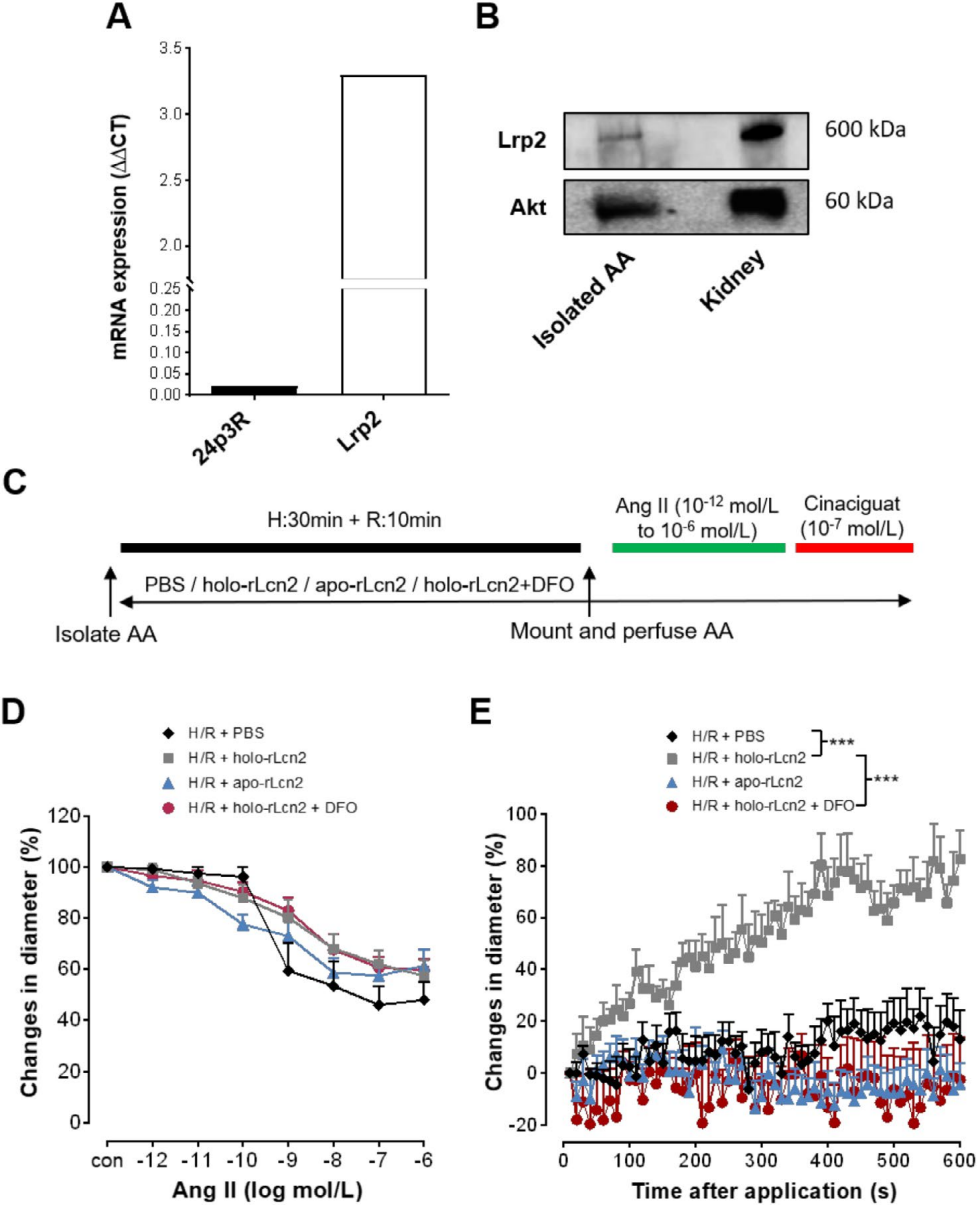
Effect of holo‐rLcn2 on Ang II‐pre‐constricted and cinaciguat treated afferent arterioles after hypoxia/reoxygenation. mRNA expression of Lcn2 receptors, low density lipoprotein‐related protein 2 (Lrp2), also known as megalin and 24p3 receptor (24p3R) in isolated afferent arterioles (AAs). 118 AAs, isolated from 12 kidneys of 6 C57BL/6 mice were pooled for RNA isolation and subsequent real‐time qPCR. Therefore, the bar graphs represent single values (A). A total of 338 AAs, isolated from 48 kidneys of 24 C57BL/6 mice, were pooled to obtain approximately 1 μg of total protein, which was entirely used for Western blot analysis. An equal amount of total protein (1 μg) isolated from mouse kidneys was used as a positive control. Since SDS‐PAGE was run longer to separate Lrp2 (~600 kDa), Akt (~60 kDa), having a relatively higher molecular weight, was used as the loading control. Representative Western blot images for Lrp2 and Akt are shown (B). The original Western blot images are presented in Figure S3. AAs were isolated from C57BL/6 mice and treated with PBS (1 μL/mL), holo‐rLcn2 (1 μg/mL), apo‐rLcn2 (1 μg/mL), or holo‐rLcn2 + (deferoxamine) DFO (100 μM) and exposed to hypoxia/reoxygenation (*H*: 30 min/*R*: 10 min). Angiotensin II (Ang II) was then applied in increasing concentrations (10^−12^ to 10^−6^ mol/L, each for 2 min) in all groups to induce vasoconstriction and recorded vascular diameter. Thereafter, dilation was induced by a bolus application of cinaciguat (10^−7^ mol/L) to the pre‐constricted AAs and their diameter was recorded every 10 s over a period of 10 min (C). Percentage changes in the microvascular diameters during Ang II‐mediated constriction (D) and cinaciguat‐mediated dilation (E) are shown. (Mean ± SEM values; *n* = 6, except *n* = 7 *H*/*R*.) ***p < 0.001 (Brunner test, Friedman test).

Since endocytic delivery of rLcn2 relies on its receptor‐mediated uptake by targeted cells, we first analyzed the expression of Lcn2 receptors (Lrp22 and 24p3R) in mouse AAs (Figure 2A). mRNA expression analysis revealed that both receptors are present in AA, with Lrp2 expressed at a higher level compared to 24p3R, suggesting a potential route for Lcn2 uptake by Lrp2 in vascular cells. The expression of Lrp2 in AA was further confirmed at the protein level by immunoblotting (Figure 2B). However, as expected, Lrp2 expression was very low in isolated AAs compared to the kidney tissue samples, which served as a positive control. To determine the effect of rLcn2 on AA function and to test if the effect depends on iron (Fe), isolated AAs were treated with carrier (PBS), holo‐rLcn2 (Fe^3+^‐bound), apo‐rLcn2 (Fe‐free) or holo‐rLcn2 + DFO (deferoxamine, iron chelator), followed by *H*/*R* (*H*: 30 min + *R*: 10 min) and analysis of vascular constriction and dilation (Figure 2C). There was no significant difference in the constriction of AAs to Ang II among different treatment groups (Figure 2D). Interestingly, while the dilation of AAs by cinaciguat was impaired due to *H*/*R*, the holo‐rLcn2 pretreatment recovered it significantly (Figure 2E). In contrast, treatment with apo‐rLcn2 did not affect the response to cinaciguat after *H*/*R*, suggesting that the effect of Lcn2 is iron‐dependent. Furthermore, pretreatment with DFO reversed the protective effect of holo‐rLcn2 treatment, validating the role of iron. Additionally, we observed that apo‐rLcn2 and holo‐rLcn2 treatments do not influence the cinaciguat‐induced dilation of AAs under normoxic conditions (Figure S4). The initial vessel diameters before Ang II‐mediated vasoconstriction, as well as immediately before cinaciguat‐mediated dilation, were comparable across treatment groups (Figure S5).

### 3.3 Holo‐rLcn2 Does Not Affect the Physiological Activation of sGC by NO

**FIGURE 3:**
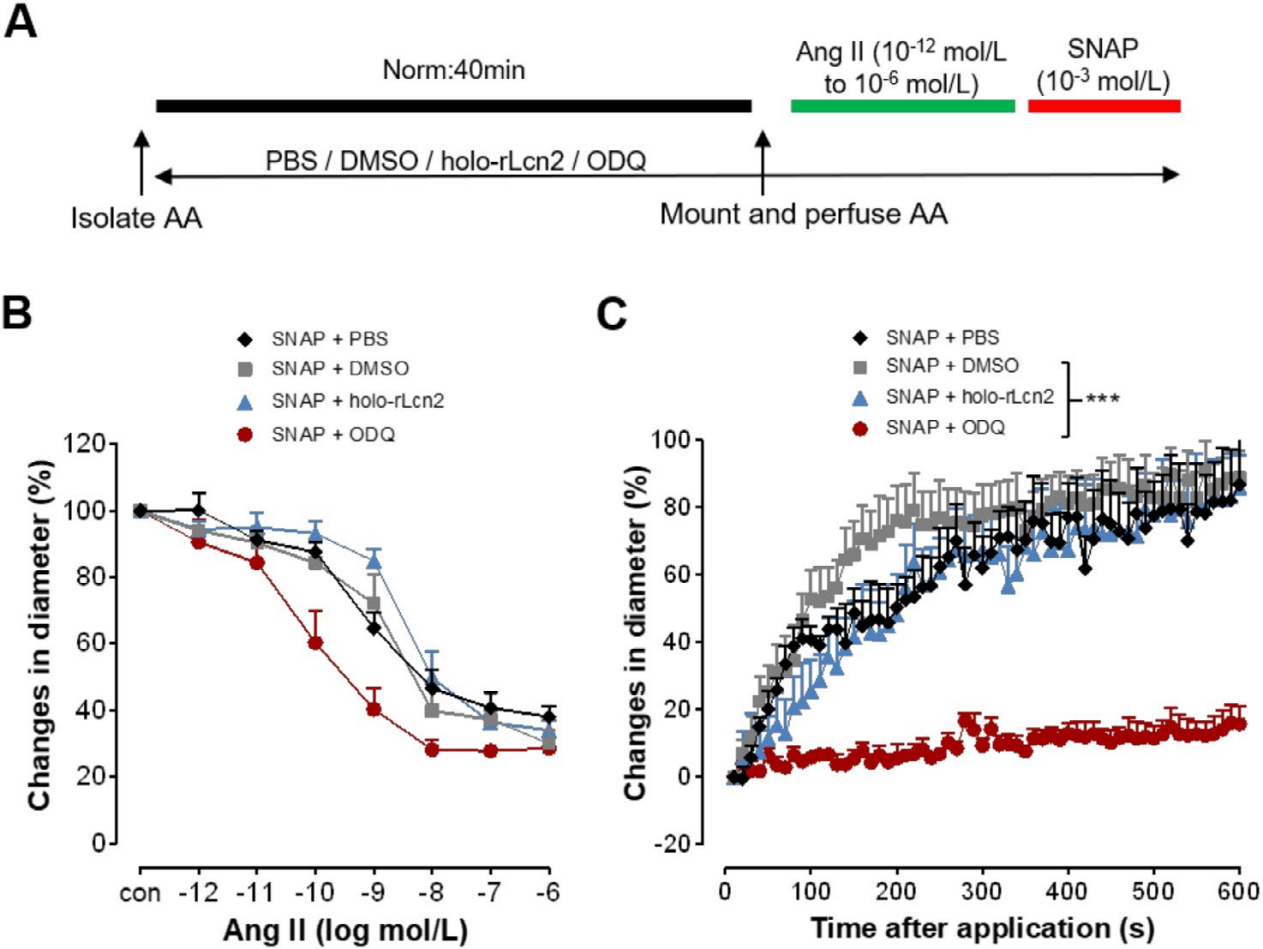
Holo‐rLcn2 does not affect SNAP‐induced dilation of Ang II‐pre‐constricted afferent arterioles under normoxia. Afferent arterioles (AAs) were isolated from C57BL/6 mice and pretreated with PBS (1 μL/mL), DMSO (1 μL/mL), holo‐rLcn2 (1 μg/mL), or ODQ (10^−5^ mol/L) and exposed to normoxia (Norm: 40 min). Angiotensin II (Ang II) was then applied in increasing concentrations (10^−12^ to 10^−6^ mol/L, each for 2 min) in all groups to induce vasoconstriction and vascular diameter was recorded. Thereafter, dilation was induced by a bolus application of SNAP (10^−3^ mol/L) to the pre‐constricted AAs and vascular diameters were recorded every 10 s over a period of 10 min (A). Percentage changes in the microvascular diameters during Ang II‐mediated constriction (B) and SNAP‐mediated dilation (C) are shown. (Mean ± SEM values; *n* = 5 PBS, *n* = 6 holo‐rLcn2, *n* = 7 DMSO, ODQ.) ***p < 0.001 (Brunner test, Friedman test).

Assuming that holo‐rLcn2 mediates dilation of AAs following *H*/*R* by favoring the oxidized state of sGC, which is better targeted and activated by cinaciguat, we questioned whether holo‐rLcn2 oxidizes sGC and interferes with the physiological activation of NO‐sGC signaling under normoxia. The isolated AAs were pretreated with vehicles (PBS, DMSO), holo‐rLcn2, or sGC oxidant ODQ (10^−5^ mol/L), followed by normoxia (40 min) and analysis of vascular constriction and dilation (Figure 3A). To mimic the physiological activation of sGC, the Ang II preconstricted AAs were treated with NO donor SNAP (10^−3^ mol/L), instead of cinaciguat. There was no significant difference in the constriction of AAs to Ang II among different treatment groups (Figure 3B). While SNAP dilated vessels to about 80% of the level before Ang II‐pre‐constriction, the sGC oxidant ODQ diminished the dilatory effect of SNAP (Figure 3C). Interestingly, holo‐rLcn2 treatment did not interfere with the SNAP‐mediated dilation of AAs, suggesting that under physiological conditions holo‐rLcn2 does not affect the activation of sGC by NO.

### 3.4 Holo‐rLcn2 Ameliorates Ischemia‐Induced Functional Impairment of Afferent Arterioles in Mouse Kidney Transplants

**FIGURE 4:**
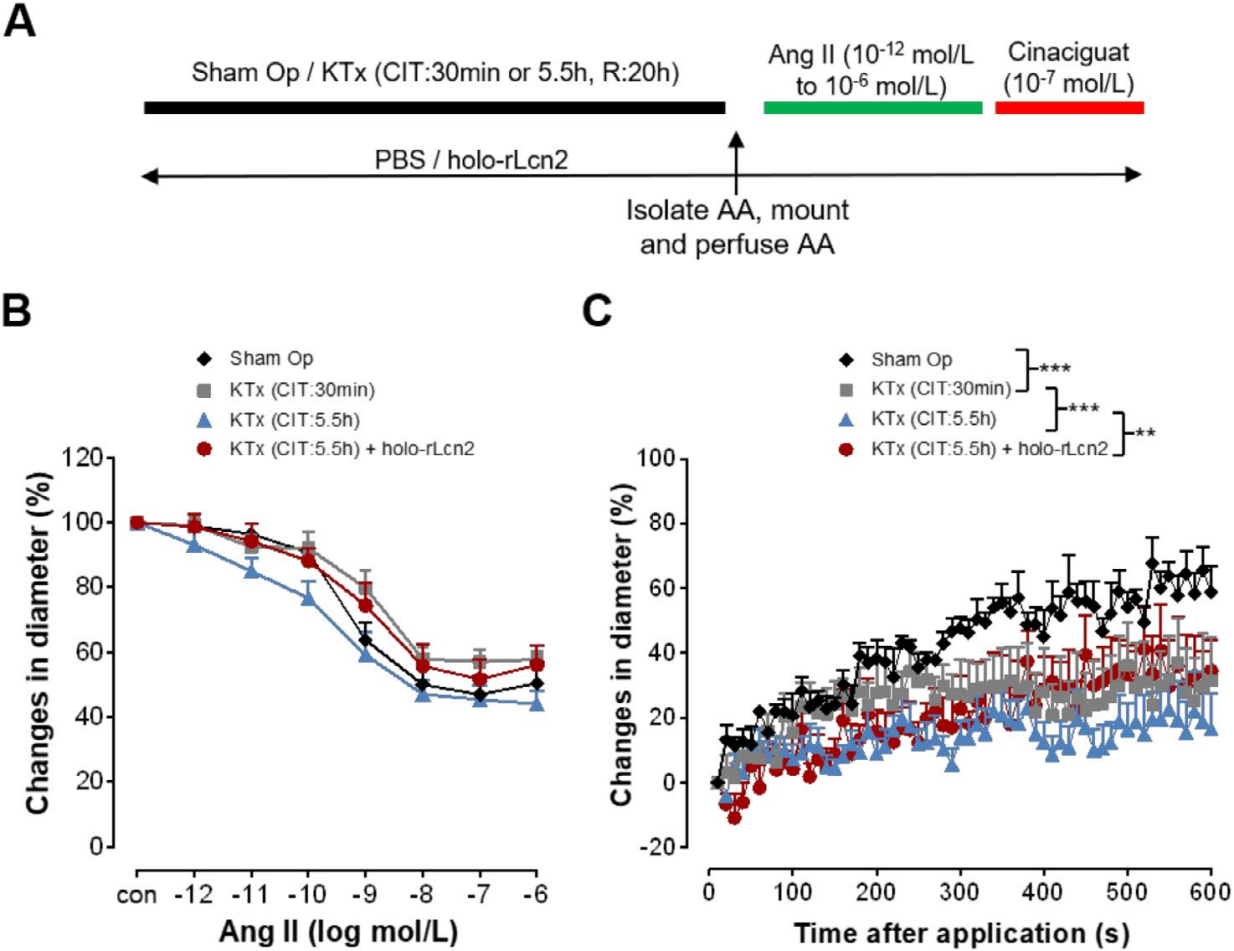
Loss in dilation of isolated afferent arterioles due to cold ischemia in kidney transplants is circumvented by treatment with holo‐rLcn2. Afferent arterioles (AAs) were isolated from sham‐operated (Sham‐Op) mouse kidneys or kidney grafts (KTx) after 20 h of syngeneic transplantation (C57BL/6 to C57BL/6), following either 30 min or 5.5 h of cold ischemia time (CIT) or 5.5 h of CIT + holo‐rLcn2 (250 μg) treatment. Angiotensin II (Ang II) was then applied in increasing concentrations (10^−12^ to 10^−6^ mol/L, each for 2 min) to the bath solution in all groups to induce vasoconstriction in AAs and vascular diameter was recorded. Dilation was induced by a bolus application of cinaciguat (10^−7^ mol/L) to the pre‐constricted AAs and vascular diameters were recorded every 10 s over a period of 10 min (A). Percentage changes in the microvascular diameters during Ang II‐mediated constriction (B) and cinaciguat‐mediated dilation (C) are shown. (Mean ± SEM values; *n* = 6 Sham‐Op, KTx [CI: 30 min], KTx [CI: 5.5 h], *n* = 9 KTx [CI: 5.5 h + holo‐rLcn2].) ***p < 0.001, **p < 0.01 (Brunner test, Friedman test).

To determine the effect of ischemia on the physiology of renal microvessels in kidney transplants, we transplanted C57BL/6 mouse kidneys syngeneically after exposing them to 30 min or 5.5 h of cold ischemia. Kidneys were harvested at 20 h post‐transplantation and subjected to ex vivo functional analysis of isolated AAs (Figure 4A). There was no significant difference in the constriction function of AAs to Ang II among the sham‐operated and kidney transplantation groups (Figure 4B). However, cold ischemia for 30 min, followed by syngeneic kidney transplantation, substantially impaired the dilatory function of AAs compared with the sham‐operated animals (Figure 4C). The longer ischemia of 5.5 h caused more severe damage, resulting in a further decrease in the dilation of AAs. Perioperative treatment with holo‐rLcn2 significantly improved the dilatory function of AAs impaired due to prolonged cold ischemia (5.5 h), restoring the dilation to a level observed for the shorter (30 min) cold ischemia group (Figure 4C).

### 3.5 Holo‐rLcn2 Improves Cinaciguat‐Induced Vascular Relaxation in the Isolated Perfused Mouse Kidneys

**FIGURE 5:**
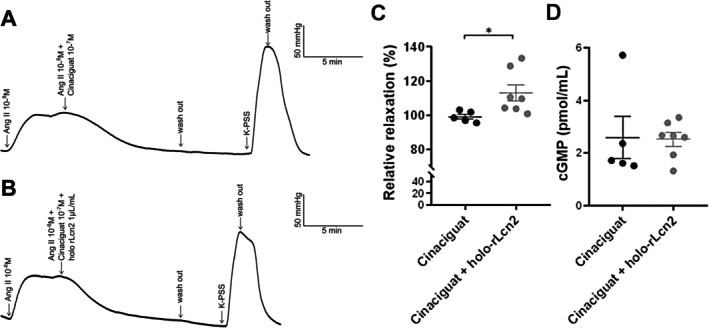
Holo‐rLcn2 enhances the vasodilatory function of cinaciguat in extracorporeal machine‐perfused isolated mouse kidneys. Kidneys of C57BL/6 mice were perfused with oxygenated physiological salt solution (PSS) at 37°C. Following an equilibration phase of 20 min, the flow rate was adjusted to raise the renal resistance to 80–90 mmHg. Then, the kidney vessels were pre‐constricted with Ang II (10^−8^ mol/L). Once a plateau was reached, either cinaciguat (10^−7^ mol/L) or a combination of cinaciguat and holo‐rLcn2 (1 μg/mL) was added to the perfusate to dilate the renal vessels. Representative pressure time curves are shown without (A) and with (B) the addition of holo‐rLcn2. The changes in perfusion pressure (Δ relaxation/Δ pre‐constriction) were used to determine the relative relaxation (C). cGMP levels were measured in the perfusates of the ex vivo perfused kidneys collected immediately after maximum relaxation was achieved (D). (Individual, mean, 25th and 75th percentile values; *n* = 5 cinaciguat, *n* = 7 cinaciguat + holo‐rLcn2.) *p < 0.05 (Welch's *t*‐test).

To evaluate whether functional improvement in the isolated AAs by holo‐rLcn2 treatment is reflected at the organ level, we performed an analysis of vascular relaxation in the intact mouse kidneys by using an isolated organ perfusion system. The kidneys were first perfused with Ang II (10^−8^ mol/L) to induce vasoconstriction, followed by vascular dilation with cinaciguat (10^−7^ mol/L) or a combination of cinaciguat and holo‐rLcn2 (1 μg/mL). Compared with the control (cinaciguat), the perfusion of the kidney with holo‐rLcn2 + cinaciguat resulted in an early drop in the perfusion pressure (Figure 5A,B), suggesting an increased dilation of renal microvessels. Interestingly, vascular relaxation relative to the Ang II‐pre‐constricted level was significantly higher in the kidneys perfused with cinaciguat + holo‐rLcn2 compared with cinaciguat alone (Figure 5C).

To investigate whether holo‐rLcn2 facilitates vascular relaxation in the isolated perfused kidney by upregulating sGC activity, we measured cGMP levels in the perfusate immediately after maximum relaxation was achieved. However, no significant differences were observed between the cinaciguat and cinaciguat + holo‐rLcn2 treated groups (Figure 5D).

## 4 Discussion

The study demonstrates that holo‐rLcn2 ameliorates the pharmacologically induced dilation of renal preglomerular vessels by the sGC activator cinaciguat after *H*/*R* and after syngeneic kidney transplantation in mice. In view of a reduced microvascular dilatory function in acute kidney injury, our findings suggest a potential protective effect of the combined application of holo‐rLcn2 and cinaciguat.

AAs contribute significantly to the total renal vascular resistance and are crucial in determining renal blood flow. In the in vitro model of *H*/*R* in mouse isolated AAs, the sGC activator‐mediated vascular dilation was significantly impaired, consistent with previous findings using a similar *H*/*R* protocol [15]. Here we show that pretreatment of AAs with holo‐rLcn2 enhances the dilatory response to cinaciguat following *H*/*R*. Thus, a combination of holo‐rLcn2 and cinaciguat may be a potent approach for restoring impaired renal perfusion in conditions of severe hypoxia and ischemia. The improved dilation response to cinaciguat following holo‐rLcn2 treatment in the isolated perfused kidney model further supports this conclusion. Additionally, holo‐rLcn2 enhanced cinaciguat‐induced dilation in AAs of transplanted kidneys subjected to prolonged cold ischemia, indicating that this drug combination may help preserve microvascular function in renal transplants.

Lcn2 is internalized by targeted cells through specialized receptors, Lrp2 and 24p3R. In the kidney, Lrp2 is primarily expressed on the apical surface of proximal tubular epithelial cells, while 24p3R is found on epithelial cells of the distal tubule and collecting duct [33]. Our data demonstrate the expression of Lcn2 receptors, especially Lrp2 in isolated AAs, suggesting a potential route for rLcn2 uptake. Presumably, holo‐rLcn2 is internalized by microvascular smooth muscle cells through receptor‐mediated endocytosis, thereby protecting microvessels from hypoxic and ischemic injury‐induced loss of dilatory function. This aligns with the protective effects observed following pre‐ and peri‐operative administration of holo‐rLcn2 in mice subjected to renal IRI, as evidenced by reduced histological damage and preserved renal function [9, 10]. Similarly, in vitro experiments confirmed that rLcn2 inhibits apoptosis in HK‐2 cells, which were exposed to 1 h of hypoxia followed by 24 h of reoxygenation [34]. Interestingly, our findings suggest that holo‐rLcn2 exerts early effects, occurring within minutes to hours. This is consistent with in vitro studies showing that rLcn2 protects HK‐2 cells from hypoxia (6 h)‐induced apoptosis by activating autophagy [35].

The protective effect of rLcn2 on renal vascular function seems to be iron dependent, as only the Fe^3+^‐bound version of rLcn2 (holo‐rLcn2) proved to be effective, while its effect was abolished by the iron chelator DFO. Likewise, other studies confirm that the protective effect of holo‐rLcn2 on renal IRI relies on its uptake and Fe^3+^ delivery to the proximal tubular epithelial cells [10]. The holo‐rLcn2 conferred protection against IRI by upregulating heme oxygenase‐1, a protective enzyme, preserving proximal tubule N‐cadherin, and inhibiting cell death [10]. Furthermore, iron may protect vascular cells from hypoxic and ischemic injury through its essential roles in oxygen transport, mitochondrial function, and antioxidant defense [36, 37, 38]. However, when in excess, iron may lead to cell death and tissue damage, mainly through favoring the generation of reactive oxygen species (ROS) [38, 39]. The protective or damaging effect of Lcn2 seems to also depend on the targeted cell type, as holo‐rLcn2 induced mitochondrial ROS generation, attenuated mitochondrial oxidative phosphorylation, and induced apoptosis in rat cardiomyocytes [40, 41]. Besides, an iron‐independent role of Lcn2 on mitochondrial dysfunction in renal tubular cells following IRI has been observed [42].

Alternatively, it can be speculated that holo‐rLcn2 enhances microvascular function during *H*/*R* and kidney transplantation by directly supporting cinaciguat‐mediated sGC‐cGMP‐PKG signaling. However, this appears less likely, as analysis of cGMP levels in the perfusate of isolated perfused mouse kidneys showed no significant difference between the cinaciguat versus cinaciguat + holo‐rLcn2 treated groups. It is also possible that holo‐Lcn2 facilitates sGC‐cGMP‐mediated dilation of AAs by targeting signaling pathways downstream of sGC. Future studies will elucidate the precise mechanisms underlying holo‐rLcn2‐mediated protection of microvascular dilation.

Interestingly, neither apo‐ nor holo‐rLcn2 affected sGC‐induced dilation under normoxic conditions, suggesting that holo‐rLcn2 exerts its effects specifically in *H*/*R*‐damaged cells. Furthermore, pretreatment with holo‐rLcn2 did not influence the dilation of AAs by NO donor SNAP, suggesting that holo‐rLcn2 does not interfere with the NO‐sGC‐cGMP‐PKG‐mediated physiological dilation of the microvessels.

## 5 Conclusion

Holo‐rLcn2 restores cinaciguat‐mediated dilation in AAs following *H*/*R* and kidney transplantation in mice, suggesting a potential protective role in kidney IRI. Furthermore, the findings highlight a potential therapeutic benefit of the combined application of holo‐rLcn2 and cinaciguat.

## Author Contributions

**Liang Zhao:** formal analysis, funding acquisition, investigation, methodology, validation, writing – original draft, writing – review and editing. **Minze Xu:** methodology, formal analysis, investigation, validation, visualization, writing – review and editing, writing – original draft. **Anna Maria Pfefferkorn:** formal analysis, investigation, methodology, validation. **Cem Erdogan:** formal analysis, investigation, methodology, validation, writing – original draft. **Hubert Schwelberger:** investigation, methodology, validation, writing – original draft. **Pinchao Wang:** conceptualization, formal analysis, methodology, investigation, validation, writing – original draft, writing – review and editing. **Pratik Hemant Khedkar:** formal analysis, investigation, methodology. **Marc Eigen:** formal analysis, investigation, methodology. **Falk‐Bach Lichtenberger:** investigation, methodology, visualization. **Rusan Catar:** formal analysis, methodology, investigation, validation, writing – review and editing. **En Yin Lai:** investigation, formal analysis, validation. **Felix Aigner:** conceptualization, funding acquisition, resources, supervision. **Pontus B. Persson:** funding acquisition, resources, supervision, writing – original draft. **Igor Maximilian Sauer:** funding acquisition, resources, supervision, writing – original draft. **Andreas Patzak:** conceptualization, investigation, funding acquisition, writing – original draft, methodology, validation, writing – review and editing, project administration, supervision, resources. **Muhammad Imtiaz Ashraf:** conceptualization, funding acquisition, investigation, methodology, project administration, resources, supervision, validation, writing – original draft, writing – review and editing, visualization, formal analysis.

## Conflicts of Interest

The authors declare no conflicts of interest.

## Supporting information


Figures S1–S5.

## Data Availability

The data that support the findings of this study are available from the corresponding author upon reasonable request.
