# Supplementary material for: Lipocalin‐2 Restores Soluble Guanylyl Cyclase‐Dependent Dilation of the Afferent Arteriole After Renal Transplantation or Ex Vivo Hypoxia/Reoxygenation in Mice
Source: Acta Physiol (Oxf). 2025 Jul 4;241(8):e70077. doi: 10.1111/apha.70077 (PMC12231162; doi:10.1111/apha.70077)
Supplement: Supplementary file 1 — Figures S1–S5. [file APHA-241-e70077-s001.docx]

**Supplementary Information**


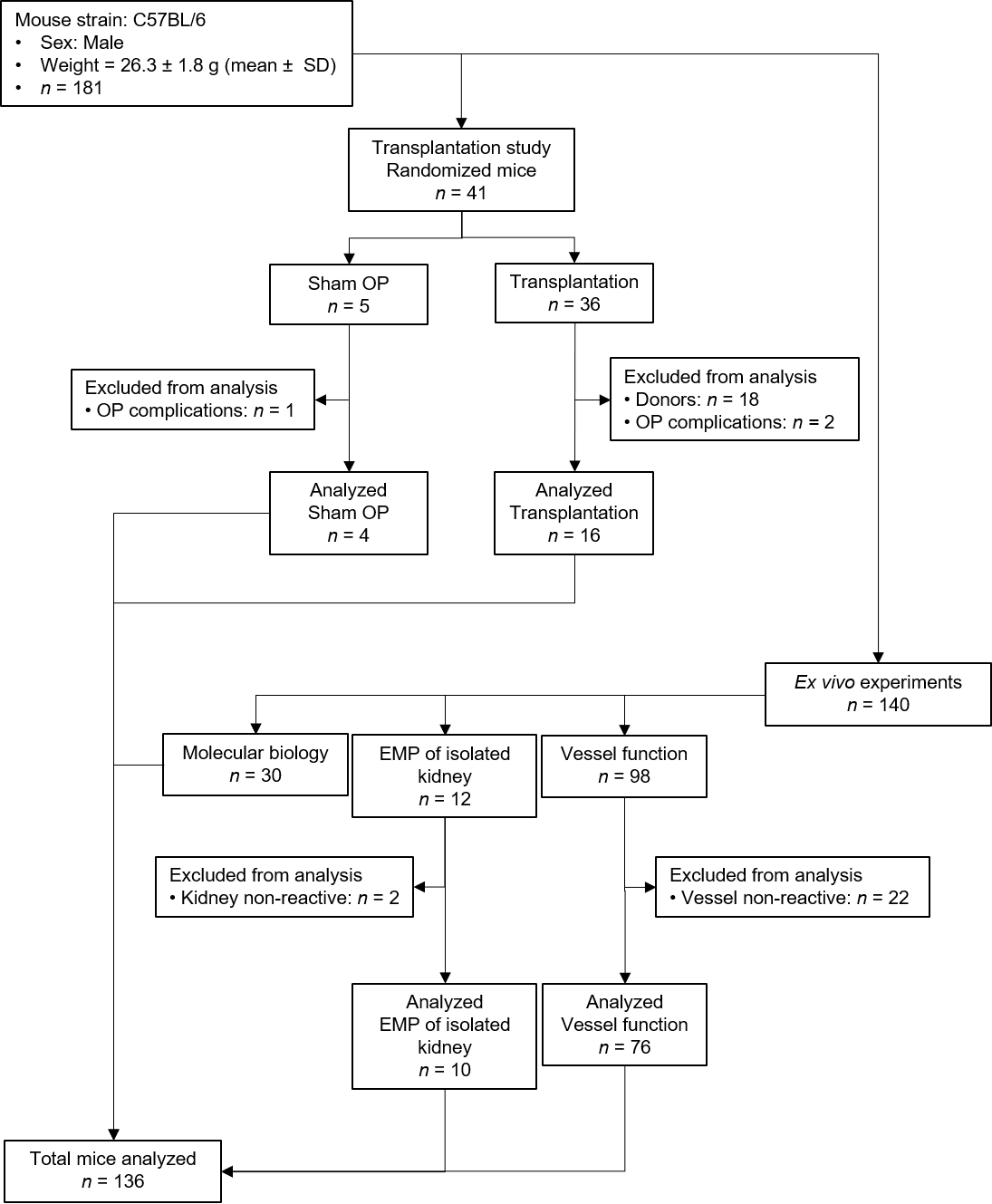


**Figure S1.** **CONSORT-like diagram showing details of the mice used.** For vessel function and EMP experiments, animals were excluded from the analysis if the isolated vessels or kidneys were non-responsive at any point during the experiment. For *in vivo* experiments (mouse kidney transplantation, sham op) donor animals and those with operational complications were excluded from the analysis. OP = operation, EMP = extracorporeal machine perfusion.


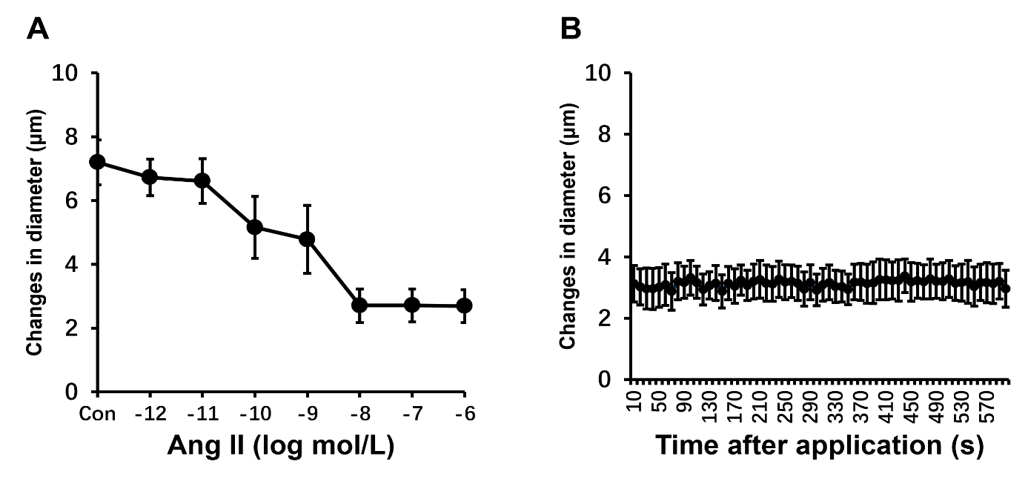
**Figure S2. Absolute lumen diameter changes in response to Ang II.** Angiotensin II (Ang II) was applied to afferent arterioles (AAs) in increasing concentrations (10^-12^ mol/L to 10^-6^ mol/L, each for 2 minutes) **(A)**. After reaching the highest concentration (10^-6^ mol/L), the vascular lumen diameter was recorded for a period 10 minutes **(B)**. (Mean ± SEM values; n=5).


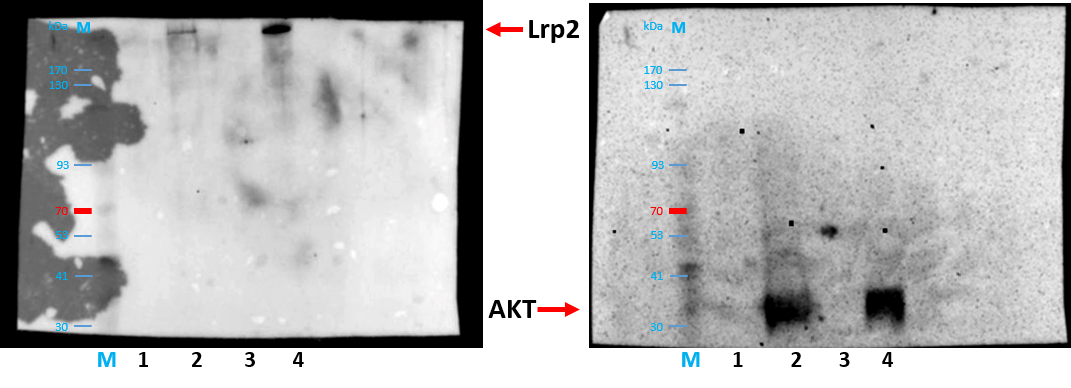


**Figure S3. The original Western blot images of the nitrocellulose membranes showing Lrp2 and Akt signals.** M=Marker (Prestained Protein Ladder – Broad molecular weight (10-245 kDa) (ab116028), 1= empty, 2=Afferent arterioles, 3=empty, 4=Kidney.


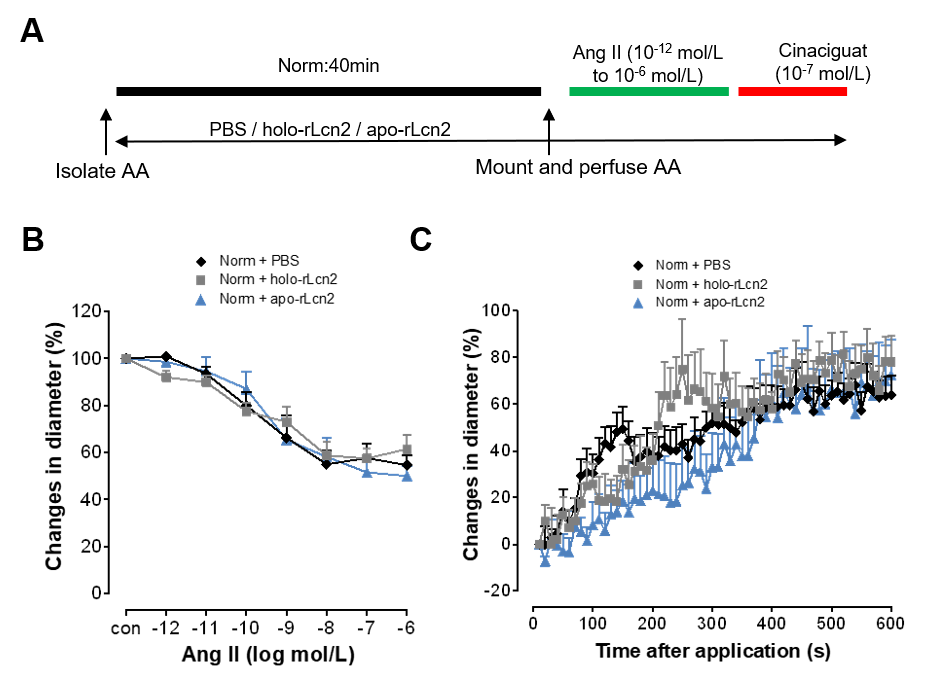


**Figure S4. Effect of holo-rcLn2 and apo-rLcn2 on Ang II-pre-constricted and cinaciguat treated afferent arterioles under normal condition.** Afferent arterioles (AAs) were isolated from C57BL/6 mice and pretreated with PBS (1 µL), holo-rLcn2 (1 µg/mL) or apo-rLcn2 (1 µg/mL). Angiotensin II (Ang II) was applied in increasing concentrations (10^-12^ mol/L to 10^-6^ mol/L, each for 2 minutes) in all groups to induce vasoconstriction and vascular diameter was recorded. Thereafter, dilation was induced by a bolus application of cinaciguat (10^-7^ mol/L) to the pre-constricted AAs and their diameter was recorded every 10 seconds over a period of 10 minutes (**A**). Percentage changes in the microvascular diameters during Ang II-mediated constriction (**B**) and cinaciguat-mediated dilation (**C**) are shown. (Mean ± SEM values; n=6 holo-rLcn2, n=8 apo-Lcn2). The change in the lumen diameter of afferent arterioles is expressed as a percentage. PBS: phosphate-buffered saline, Norm: normoxia.


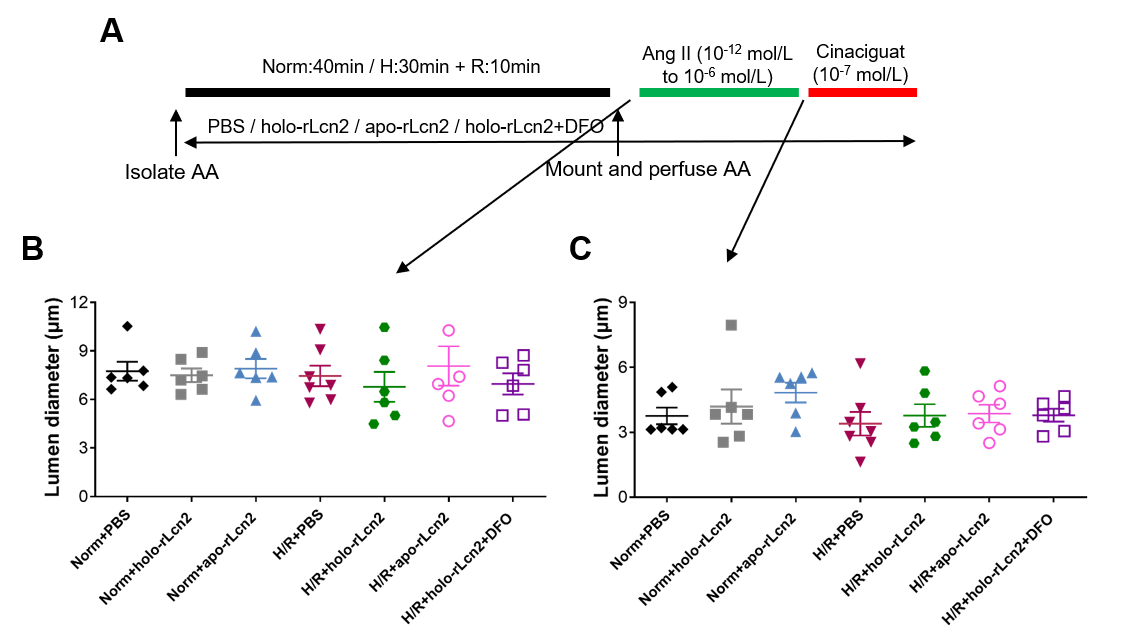


**Figure S5. Absolute lumen diameters of isolated afferent arterioles before Ang II-mediated vasoconstriction and before cinaciguat-mediated vasodilation.** Afferent arterioles (AAs) were isolated from C57BL/6 mice, pretreated with PBS (1 µL/mL), holo-rLcn2 (1 µg/mL), apo-rLcn2 (1 µg/mL) or holo-rLcn2 + DFO (100 µM) and exposed to hypoxia/reoxygenation (H: 30 minutes/ R: 10 minutes) or normoxia for the same duration. Angiotensin II (Ang II) was then applied in increasing concentrations (10^-12^ mol/L to 10^-6^ mol/L, each for 2 minutes) to the bath solution in all groups to induce vasoconstriction in AAs and vascular diameter was recorded. Dilation was induced by a bolus application of cinaciguat (10^-7^ mol/L) to the pre-constricted AAs and vascular diameters were recorded every 10 seconds over a period of 10 minutes (**A**). Diameters of the AAs were recorded in each group before the first dose of angiotensin II (**B**), as well as immediately before bolus application of cinaciguat (**C**). (Mean ± SEM values; n=6-7). Norm: normoxia, H/R: hypoxia/reoxygenation, DFO: deferoxamine mesylate.
